# Overview of microbial profiles in human hepatocellular carcinoma and adjacent nontumor tissues

**DOI:** 10.1186/s12967-023-03938-6

**Published:** 2023-02-02

**Authors:** Yuting He, Qiyao Zhang, Xiao Yu, Shuijun Zhang, Wenzhi Guo

**Affiliations:** 1 Department of Hepatobiliary and Pancreatic Surgery, The First Affiliated Hospital of Zhengzhou University, Zhengzhou, 450052 China; 2 Key Laboratory of Hepatobiliary and Pancreatic Surgery and Digestive Organ Transplantation of Henan Province, The First Affiliated Hospital of Zhengzhou University, Zhengzhou, 450052 China; 3 Open and Key Laboratory of Hepatobiliary & Pancreatic Surgery and Digestive Organ Transplantation at Henan Universities, Zhengzhou, 450052 China; 4 Henan Key Laboratory of Digestive Organ Transplantation, Zhengzhou, 450052 China

**Keywords:** Hepatocellular carcinoma, Intratumoral microbiota, Metabolic pathway, Fatty acid and lipid synthesis, MiSeq

## Abstract

**Background:**

Intratumoral microbial communities have been recently discovered to exist in a variety of cancers and have been found to be intricately involved in tumour progression. Therefore, investigating the profiles and functions of intratumoral microbial distribution in hepatocellular carcinoma (HCC) is imperative.

**Methods:**

To verify the presence of microorganisms in HCC, we performed fluorescence in situ hybridization (FISH) using HCC tissues and conducted MiSeq using 99 HCC and paracancerous tissues to identify the key microorganisms and changes in metabolic pathways affecting HCC progression through a variety of bioinformatics methods.

**Results:**

Microbial diversity was significantly higher in HCC tissues than in adjacent tissues. The abundances of microorganisms such as *Enterobacteriaceae*, *Fusobacterium* and *Neisseria* were significantly increased in HCC tissues, while the abundances of certain antitumour bacteria such as *Pseudomonas* were decreased. Processes such as fatty acid and lipid synthesis were significantly enhanced in the microbiota in HCC tissues, which may be a key factor through which intratumoral microbes influence tumour progression. There were considerable differences in the microbes and their functions within tumour tissue collected from patients with different clinical features.

**Conclusion:**

We comprehensively evaluated the intratumoral microbial atlas of HCC tissue and preliminarily explored the mechanism of the effects of the microbial community involving changes in lipid metabolism and effects on HCC progression, which lays the foundation for further research in this field.

**Supplementary Information:**

The online version contains supplementary material available at 10.1186/s12967-023-03938-6.

## Introduction

Hepatocellular carcinoma is the sixth most common malignancy and the third leading cause of cancer-related deaths worldwide, mainly due to liver cirrhosis caused by hepatitis virus, alcohol and fat accumulation [1, 2]. As a cancer with a progressive disease course, without effective early intervention, the disease can cause an irreversible damage and extremely poor prognosis [3, 4]. Despite the great progress achieved in various treatments, such as surgery, chemoradiotherapy, immunotherapy and targeted therapy, in the past few decades, the prognosis of HCC patients is still not optimistic due to the high metastasis rate and recurrence rate of this tumour type [5–7]. Therefore, it is urgent to explore the molecular mechanisms that occur during the pathogenesis and progression of HCC.

The interior of tumours was originally thought to be sterile, especially in solid tumours. However, recent evidence suggests that intratumoral microbes form an important component of the tumour microenvironment (TME) and that these microbes are intricately involved in tumorigenesis, progression, and sensitivity to therapy in the local environment [8]. Deborah Nejman comprehensively identified the presence of abundant intratumoral bacteria in breast, lung, ovarian, pancreatic, melanoma, bone, and brain tumours. The results revealed a tumour-specific microbial composition, and the metabolic pathways and clinical features of these microorganisms were closely related [9]. At present, it is believed that intratumoral microorganisms affect tumour progression mainly by causing DNA damage, the activation of oncogenic pathways, and the regulation of the immune system in the microenvironment [10, 11]. The above results show that investigating the importance of intratumoral bacteria is an emerging field, attracting the interest of researchers for its potential role as an intervention target in tumour diagnosis and therapy. It is reasonable to speculate that microorganisms within HCC can also participate in the progression and metastasis of HCC through the above pathways. However, few relevant studies have explored the tumour microbiome in HCC. Therefore, it is necessary and meaningful to investigate the presence, abundance and functions of intratumoral microorganisms in this cancer type.

In this study, we first performed fluorescence in situ hybridization (FISH) using HCC tissues to verify the presence of bacteria in HCC, followed by MiSeq using 99 HCC tissues and adjacent tissues, to comprehensively analyse microbial infiltration and changes in the metabolic pathways that occur in HCC. Some valuable conclusions were revealed. For instance, microbes in tumours may support tumour cell proliferation and invasion through increased fatty acid and lipid synthesis. In addition, certain microorganisms may be involved in the process of HCC in unique ways. Taken together, the results suggest that intratumoral microbes interact and complement tumour cells and the TME. We believe that our research supports further developments in this field.

## Methods

### Sample collection

We collected 99 HCC and paracancerous tissue samples from patients at the First Affiliated Hospital of Zhengzhou University (Zhengzhou, Henan, China). All studies using these samples were approved by the Ethics Committee of the First Affiliated Hospital of Zhengzhou University. The average age of patients included in the study was 54.19. 71.4% patients had HBV infection. 77.3% of the patients were single tumor, and 79.1% of the patients were classified as Child A. More detailed information was shown in Table 1. All tumor tissues and adjacent tissues are obtained by surgical resection.

**Table 1:** Clinical characteristics of patients included in this study

Clinical features	Overall	Clinical features	Overall
n	44	Grade = G3 (%)	7 (22.6)
Age (mean (SD))	54.19 (9.02)	BCLC (%)	
HBV = YES (%)	30 (71.4)	A	2 (5.0)
Child (%)		A1	19 (47.5)
ChildA	34 (79.1)	A2	2 (5.0)
ChildB	6 (14.0)	A4	3 (7.5)
ChildC	3 (7.0)	B	11 (27.5)
Tumor number = single (%)	34 (77.3)	D	3 (7.5)
T stage (%)		Stage (%)	
T1	33 (75.0)	I	32 (72.7)
T2	8 (18.2)	Ib	2 (4.5)
T3	2 (4.5)	II	9 (20.5)
TI	1 (2.3)	IIIA	1 (2.3)
CA125-B (mean (SD))	53.58 (161.87)	CEA-B (mean (SD))	2.76 (2.61)

### Fluorescence in situ hybridization

Tissues were prepared into paraffin sections, added to prehybridization solution, and incubated at 37 °C for 1 h. Then, hybridization solution containing the EUB338 probe (Servicebio, G3016-3) was added, and samples were incubated at 42 °C overnight in an incubator and photographed under a microscope (NIKON DS-U3).

The probe sequence used was EUB338:*5'-CY3-GCT GCC TCC CGT AGG AGT-3'*.

### Bacterial DNA extraction and sequencing

All of the samples were subjected to the same procedures for DNA extraction and PCR amplification by the same laboratory staff. Each sample was suspended in 790 μL of sterile lysis buffer (4 M guanidine thiocyanate; 10% n-lauroyl sarcosine; 5% n-lauroyl sarcosine-0.1 M phosphate buffer [pH 8.0]) in a 2-mL screw-cap tube containing 1 g glass beads (0.1 mm BioSpec Products, Inc., USA). This mixture was vortexed vigorously and incubated at 70 °C for 1 h. After incubation by bead beating for 10 min at maximum speed, DNA was extracted using the manufacturer’s instructions for bacterial DNA extraction using the E.Z.N.A.^®^Stool DNA Kit (Omega Biotek, Inc., GA), with the exception of lysis steps, and the product was stored at − 20 °C until further analysis. The extracted DNA obtained from each sample was used as the template to amplify the V3 ~ V4 region of 16S rRNA genes.

The primers F1 and R2 (5′-CCTACGGGNGGCWGCAG-3′ and 5′-GACTACHVGGGTATCTAATCC-3′) corresponding to positions 341 to 805 in the *Escherichia coli* 16S rRNA gene were used to amplify the V3 ~ V4 region of each sample by PCR. The PCR experiments were run in an EasyCycler 96 PCR system (Analytik Jena Corp., AG, Germany) using the following program: 3 min of denaturation at 95 ℃ followed by 21 cycles of 0.5 min at 94 ℃ (denaturation), 0.5 min of annealing at 58 ℃, and 0.5 min at 72 ℃ (elongation), with a final extension at 72 ℃ for 5 min. The products from different samples were indexed and mixed at equal ratios for sequencing using the MiSeq platform (Illumina Inc., USA) according to the manufacturer’s instructions.

### Sequencing data processing and OTU (Operational Taxonomic Unit) cluster annotation

Paired-end sequence data were obtained based on MiSeq sequencing. According to the complementary region (overlap) between the PE (paired end) reads, the paired reads were merged into a single sequence. Quality control filtering was performed on data regarding the quality of reads and the effect of merging. Samples were distinguished according to the index sequences and primer sequences at both ends of the sequence to obtain high-quality effective sequences and to correct the sequence orientation.

OTU (operational taxonomic unit) is a label artificially set for a taxonomic unit (strain, genus or species) to facilitate analysis in population genetics research [12]. To identify the number of species, genera and other information in the sequencing results obtained from a sample, after removing the single sequences without repeats, we classified the sequences into taxonomic units, namely, OTUs, based on a similarity value of 97%. Chimeric sequences were removed during the clustering process to obtain the representative sequence of an OTU. Subsequently, by aligning the 16S bacterial and archaeal ribosome databases Silva3 (Release 138 http://www.arb-silva.de) [13], we performed OTU species annotation based on the QIIME platform (http://qiime.org/scripts/assign_taxonomy.html). The RDP classifier Bayesian algorithm was used to perform taxonomic analysis on the representative sequences of OTUs with a similarity level of 97%, and the community composition of each sample was counted at each classification level (phylum, class, order, family, genus, and species) [14].

### Bioinformatics analysis

Alpha diversity was defined by the Chao, Ace, Shannon, and Simpson indices, which were calculated using mothur (version v.1.42.1, http://www.mothur.org) [15]. Beta diversity was assessed by unweighted and weighted UniFrac distance matrices and visualized by principal coordinate analysis (PCoA). QIIME software was used to calculate the beta diversity distance matrix, and the R software ‘vegan’ package was employed for PCoA analysis and visualization.

LEfSe is a data analysis method based on linear discriminant analysis (LDA) effect size [16]. Specifically, we first used the nonparametric factorial Kruskal‒Wallis (KW) sum-rank test to establish differential microbiota. Next, linear discriminant analysis (LDA) was applied to estimate the magnitude of the role of species abundance in the differential effect. The algorithm emphasizes statistical significance and biological relevance. LEfSe analysis was performed with a web-based tool (http://huttenhower.sph.harvard.edu). In this study, LDA > 2.5 was considered statistically and biologically significant. Subsequently, using Qiime software (http://qiime.org), a random forest algorithm was used to identify OTUs with significant differences between groups, with 1000 trees for modelling and fivefold cross-validation to estimate the size of generalization error. The default settings were used for the rest of the parameters. All biochemical index data were obtained using the last blood sample collected from the patient before surgery. Any correlations with the abundance of microorganisms were established by Spearman’s method. Due to the small number of patients with Child‒Pugh scores B and C, we pooled the two groups.

## Results

### Fluorescence in situ hybridization (FISH) confirmed the presence of bacteria in hepatocellular carcinoma

**Fig. 1:**
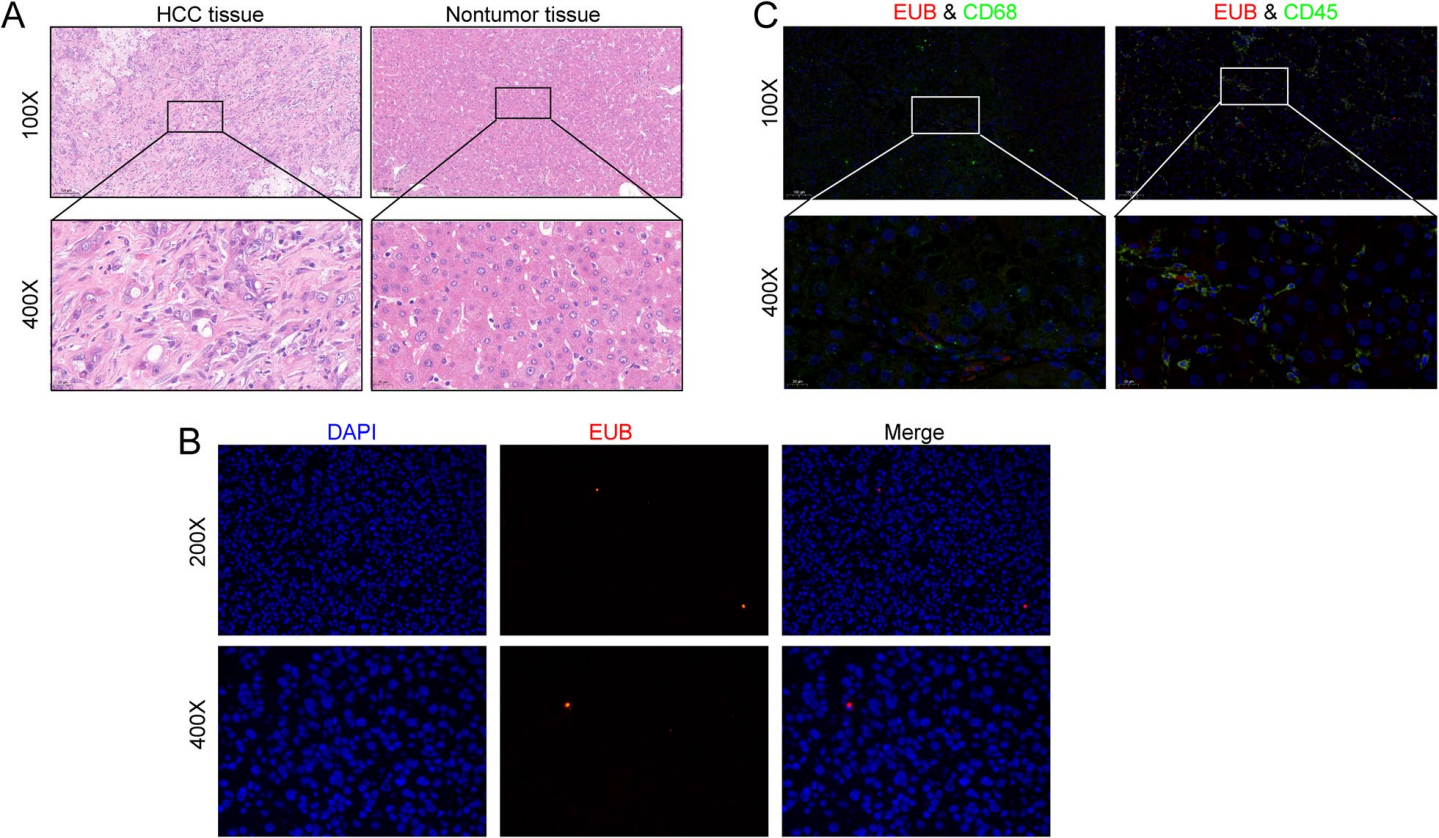
HCC tissue and immune cells in the presence of bacteria. **A** H&E staining of HCC tissue and paracancerous tissue. **B** FISH analysis of bacterial 16S rRNA sequences confirmed the presence of bacteria in HCC. **C** Representative immune cell staining. Bacteria were found in CD68 + cells and CD45 + cells. Among them, CD68 + cells are considered macrophages

We performed haematoxylin and eosin (H&E) staining on the collected tumour and nontumor tissues and evaluated the typical properties of these HCC and nontumor tissues (Fig. 1A). First, we carried out FISH detection in HCC tissues. The results confirmed that HCC tissues contained bacteria, albeit in small numbers (Fig. 1B). Furthermore, we explored the relationship between immune cells and bacteria. Using a fluorescent costaining technique, we stained for the two immune cell biomarkers CD45 and CD68, as well as EUB338, in HCC tissues. As shown in Fig. 1C, some CD68 + cells harboured bacteria, which may have been related to phagocytosis by macrophages. CD45 + cells also contained bacteria. Since these results showed the presence of bacteria in HCC tissues and immune cells in the TME, we attempted to further reveal the differences in bacterial species and abundance in HCC and paracancerous tissues by Illumina sequencing technology.

### Differences in microbiota composition between HCC and paracancerous tissues

**Fig. 2:**
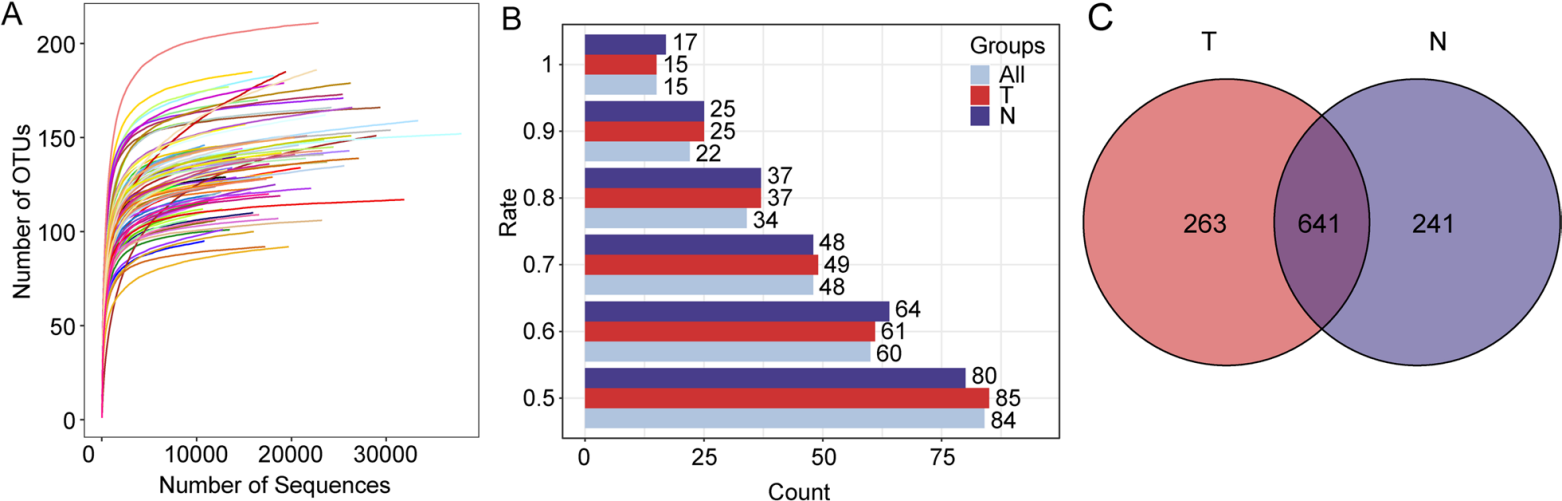
General characteristics of the microbiota in HCC and adjacent tissues. **A** Sparsity curves for sequencing data. **B** Core microbes at different sample coverages in HCC and paracancerous tissues. **C** Set-distribution analysis of OTU numbers between groups

Following the comparison of MiSeq data with the 16S bacterial and archaeal ribosome database, we identified a total of 1145 OTUs, including 28 phyla, 47 classes, 133 orders, 239 families, and 534 genera (Additional file 1: Table S1). First, a rarefaction curve analysis was performed, which is a curve generated by the number of sequences and the number of species. The results showed that at a specific sequencing depth, the curve tended to be flat, that is, an increased volume of data generated relatively few new OTUs, indicating that our sequencing data depth was reasonable (Fig. 2A). Subsequently, to evaluate the core components of the microbiome, we performed a statistical analysis at different sample coverages based on the number of shared OTUs in the samples (Fig. 2B, Additional file 1: Table S2). The findings showed that the number of shared microorganisms in the respective samples obtained from HCC and paracancerous tissue was consistent at different levels of sample coverage. Fifteen core microorganisms were identified in all samples and were consistent between HCC and paracancerous tissues. In addition, the set-distribution analysis revealed 263 unique OTUs in tumour tissue and more than 241 in adjacent tissue, indicating a significant difference in microbial species between the two groups (Fig. 2C).

### Alpha and beta diversity differences between groups

**Fig. 3:**
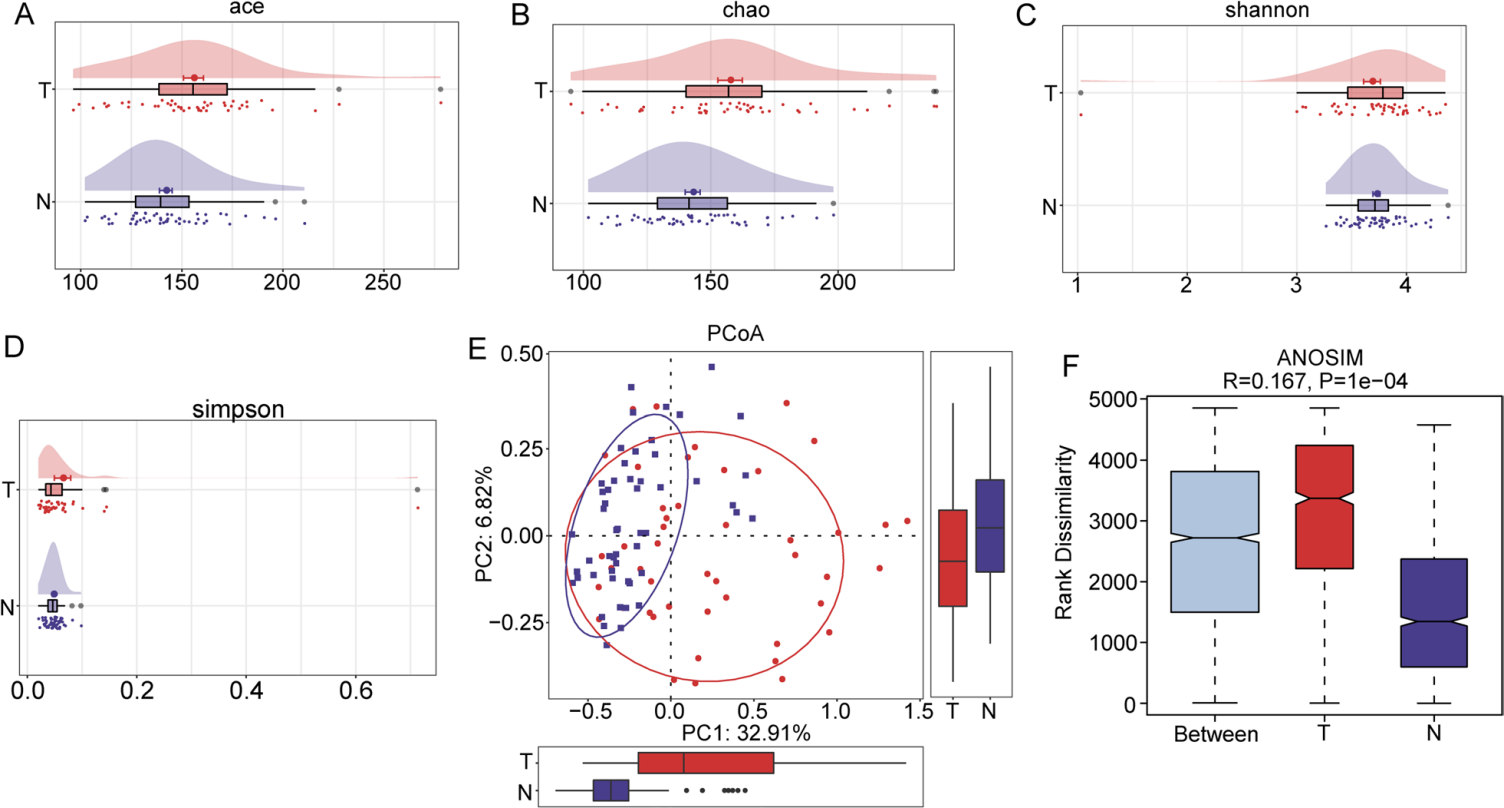
Alpha and beta diversity among microbes in HCC and paracancerous tissues. **A** Differences in the Ace index between groups. **B** Differences in the Chao index between groups. **C** Differences in the Shannon index between groups. **D** Differences in the Simpson index between groups. **E** PCoA of bacterial beta diversity based on unweighted UniFrac distances. **F** Microbiota beta diversity based on ANOSIM

Single-sample diversity analysis (alpha-diversity) was employed to describe the diversity of bacterial species and abundance in a single sample. We compared the alpha diversity between HCC and paracancerous tissues based on 4 well-established indices. The results showed that, among the various alpha diversity indices, the Ace and Chao indices were significantly higher in HCC tissues than in paracancerous tissues (Fig. 3A, B, P < 0.05), indicating that HCC samples had significantly more bacterial species than paracancerous tissues. The Simpson and Shannon indices also showed this trend, albeit without a statistically significant difference, which may have been due to the insufficient sample size (Fig. 3C, D). Beta diversity is a comparative analytical measure of the microbial community composition of samples obtained from different groups. In this study, β diversity was calculated using principal coordinates analysis and ADONIS. As presented in the figure, the PCoA results based on the unweighted UniFrac distance showed that there was a certain overall difference in the microflora in HCC and paracancerous tissues, and the results of ADONIS also uncovered a significant difference between the two groups (Fig. 3E, F, P < 0.05). The above results indicated that there were significant differences between HCC and paracancerous tissues in terms of the bacterial richness of a single sample and the overall composition of the two groups.

### Differences in microbiota composition between HCC and paracancerous tissues

**Fig. 4:**
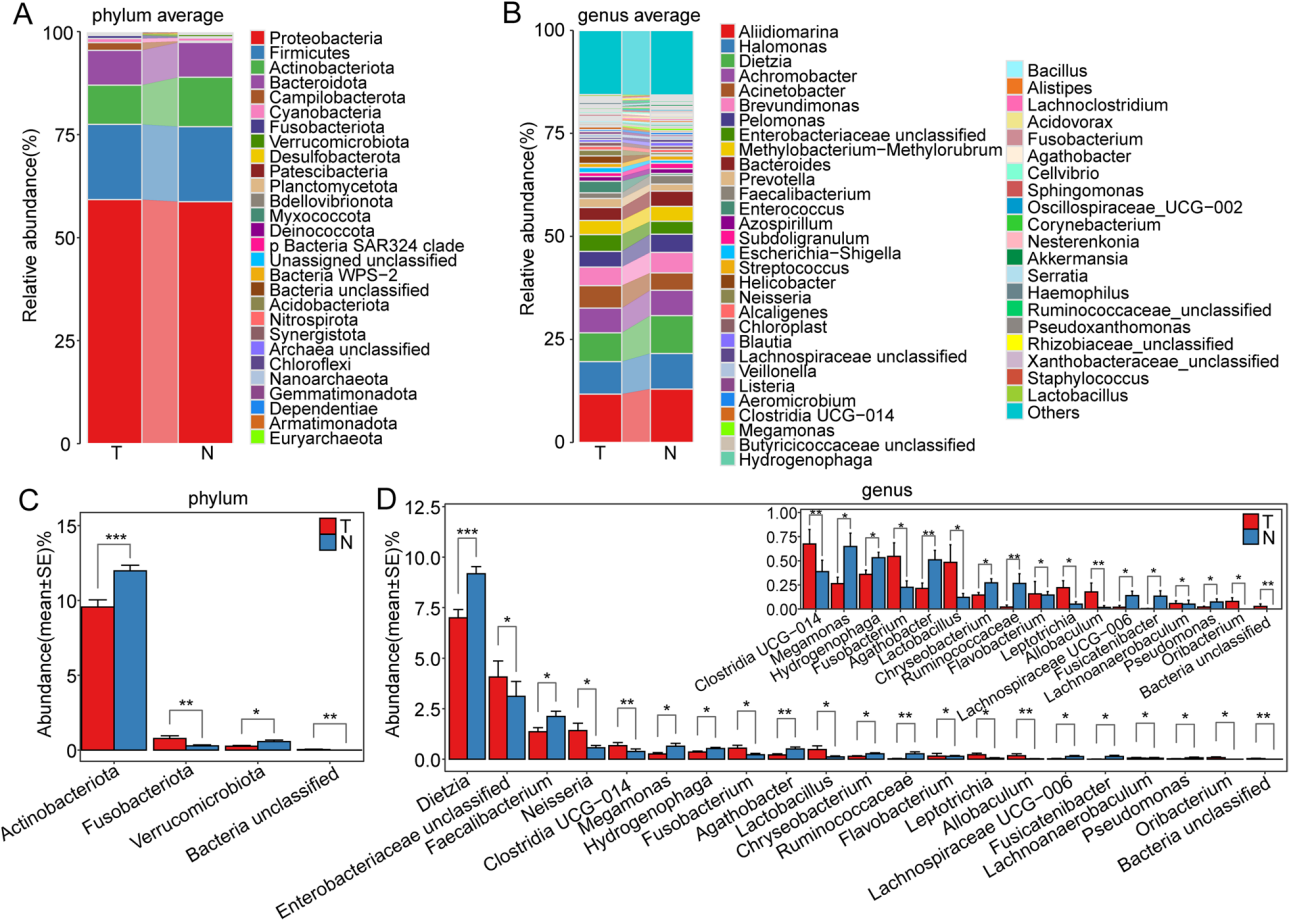
Differences in microbial composition between HCC and paracancerous tissues. **A** Microbial composition of HCC and paracancerous tissues at the phylum level. **B** Microbial composition of HCC and paracancerous tissues at the genus level. **C** Differences in microbes between study groups at the phylum level based on the Mann–Whitney U test. **D** Differences in microbes between study groups at the genus level based on the Mann‒Whitney U test

The predominant microflora in HCC and adjacent tissues were very similar. At the phylum level, the most dominant genera that differed between the two groups were Proteobacteria, followed by Firmicutes, Actinobacteriota, and Bacteroidetes. Specifically, the proportion of Proteobacteria in HCC was slightly higher, while the abundance of Actinobacteriota was significantly lower (Fig. 4A). At the genus level, *Aliidiomarina*, *Halomonas*, *Dietzia*, and *Achromobacter* were the dominant genera in the two groups. The proportion of *Alidiomarina* was lower in HCC samples (Fig. 4B).

We chose the Mann‒Whitney U test for the statistical analysis of the differential microbes between groups. According to the results, at the phylum level, Actinobacteriota and Verrucomicrobiota genera had significantly lower abundance in HCC tissues than in paraneoplastic tissue, whereas Fusobacteriota abundance followed the opposite trend (Fig. 4C). At the genus level, the abundances of the genera *Dietzia*, *Faecalibacterium*, *Megamonas*, *Hydrogenophaga*, *Agathobacter*, *Chryseobacterium*, and *Ruminococcaceae* were significantly lower, while the abundances of *Neisseria*, Clostridia_UCG-014, *Fusobacterium*, and *Lactobacillus* were significantly higher in HCC tissues than in paracancerous tissues (Fig. 4D,  P< 0.05).

**Fig. 5:**
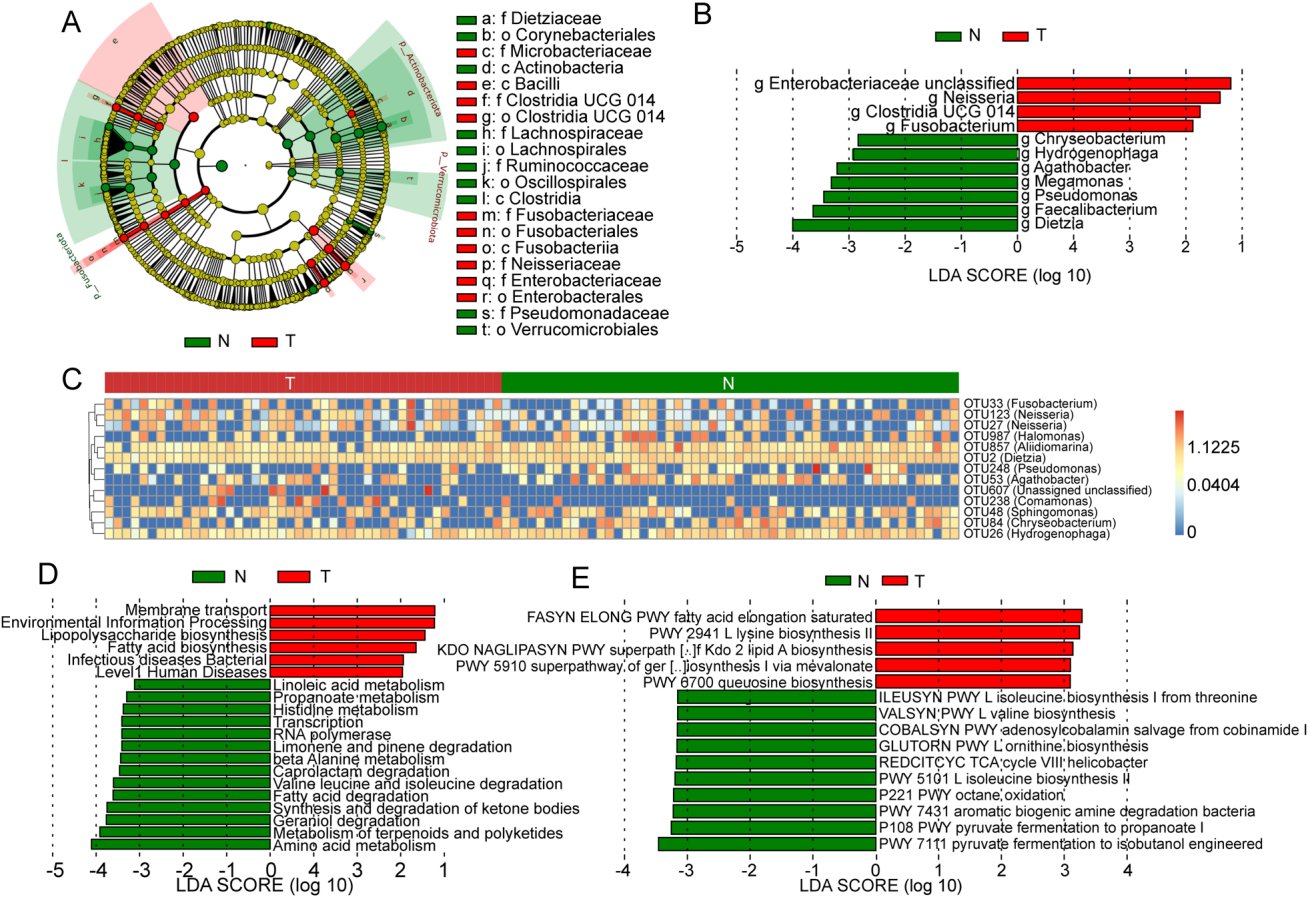
Differences in the microbiome and its function between HCC and paracancerous tissues identified based on LEfSe and random forest. **A** Clade map containing differential bacterial taxa from the phylum to the genus level. **B** LDA histogram of differential microbiota at the genus level. **C** Microbes with the largest contribution to group differences identified based on the random forest algorithm. **D** Differential metabolic pathways between groups based on the KEGG database. **E** Differential metabolic pathways between groups based on the MetaCyc database

Subsequently, LEfSe analysis was performed to identify bacterial groups with significantly different effects on sample partitioning. We found that the abundances of microbes within the same phylum generally showed the same trend of change among the study groups (Fig. 5A). Specifically, the abundances of Clostridia and Actinobacteriota tended to rise significantly in paracancerous tissue. At the genus level, using an LDA score greater than 2.5 as the threshold, we identified 11 bacterial genera that had significantly different abundances between the T and N groups (P < 0.05, LDA > 2), and the abundances of four of these genera were significantly elevated in HCC. *Enterobacteriaceae*, *Neisseria* and *Fusobacterium* had significantly higher abundances in tumour tissue. Moreover, *Chryseobacterium*, *Hydrogenophaga*, *Agathobacter*, *Megamonas*, *Pseudomonas*, *Faecalibacterium*, and *Dietzia* abundances were elevated in paracancerous tissues (Fig. 5B).

To investigate the complex nonlinear interdependencies among microbes, we identified the key microbes that differed between the two groups of samples through random forest analysis and visualized the differences in heatmaps. The results showed that 12 OTUs (containing 11 species of bacteria) were different between the two groups. Consistent with the above results, seven OTUs were low in HCC tissue, which belonged to *Sphingomonas*, *Chryseobacterium*, *Hydrogenophaga*, *Aliidiomarina*, *Dietzia*, *Pseudomonas*, and *Agathobacter*. Five OTUs had high abundance in HCC tissue, namely, *Fusobacterium*, *Neisseria* and *Comamonas,* and one unclassified species (Fig. 5C).

### Microbes in HCC tissue cause diverse changes in biological function

The microbial community in solid tumours interacts with tumour cells mainly through the metabolites they release, causing changes in cancer metabolic pathways, the regulation of immune responses in the TME, and even the occurrence of DNA damage, which in turn affects tumour progression. Understanding the microbial metabolic functions in HCC and their impact on tumour cells has important implications for investigating how microbes influence HCC progression. In this study, we aimed to investigate this process at multiple levels via PICRUSt2 prediction. The KEGG pathway analysis showed that membrane transport, fatty acid and lipopolysaccharide biosynthesis, and bacteria and disease pathways were significantly enriched in HCC, while fatty acid degradation and the metabolic processes of linoleic acid, propionate and histidine were significantly inhibited (Fig. 5D). The MetaCyc-based metabolic pathway prediction results indicated that the biosynthesis and saturated fatty acid elongation pathways of molecules such as lysine, lipid and mevalonate were significantly enhanced in HCC, while the degradation of molecules such as purines and nucleotides, glyoxylate bypass and arginine biosynthesis was inhibited (Fig. 5E). Overall, despite differences in predictions from different metabolic function databases, the key pathways involved in the impacts of the microbiota on tumour cells in HCC appear to be fatty acid and lipid biosynthesis, metabolic inhibition of small molecules, and amino acid imbalances.

### Differences in the intratumoral microbiome are significantly associated with clinical features

**Fig. 6:**
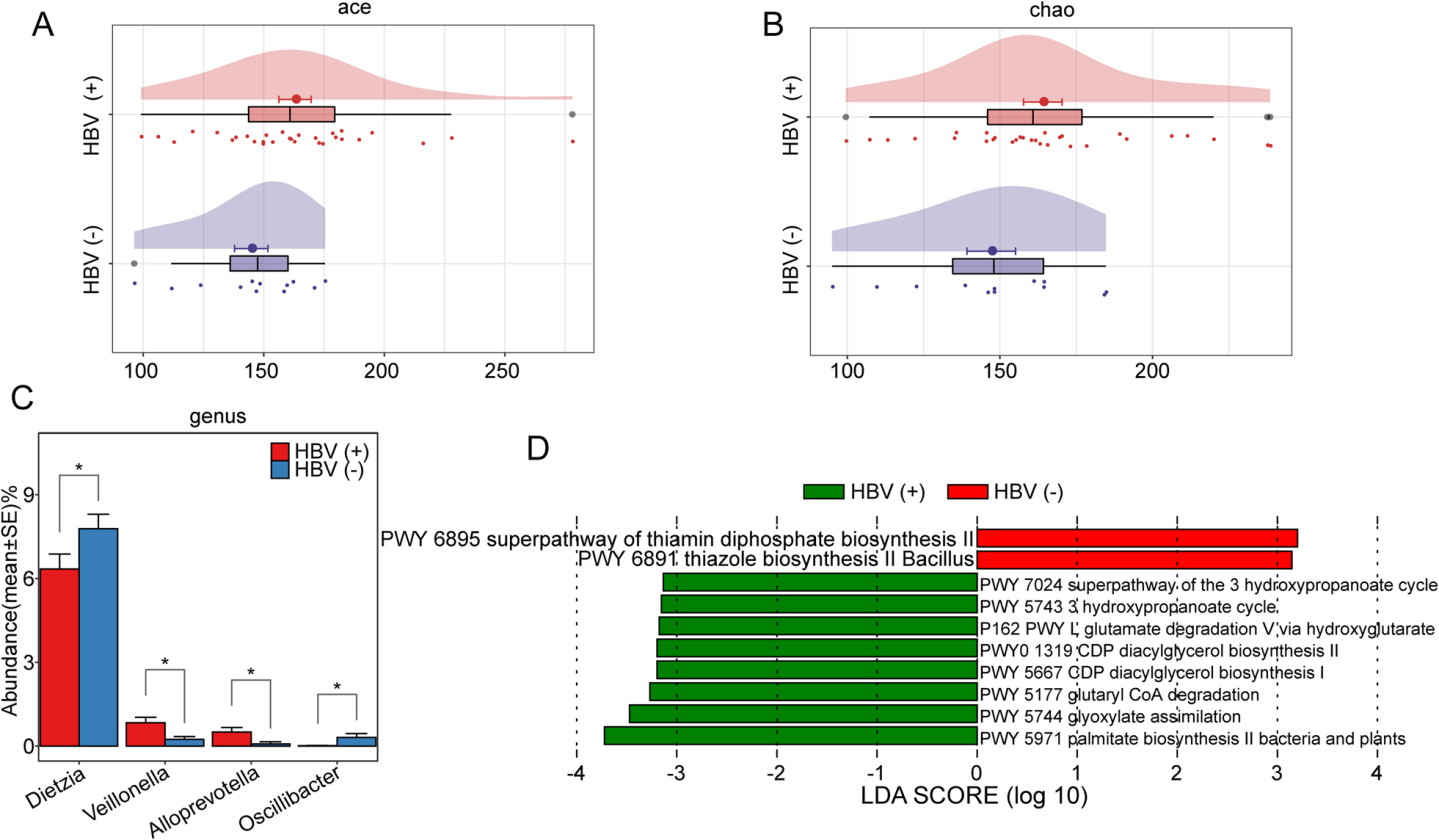
Differences in the microbiota of HBV-related and non-HBV-related HCC. **A**, **B** Differences in the Ace and Chao indices of microbiota between HBV-related and non-HBV-related HCC. **C** Microbial differences at the genus level between HBV-related and non-HBV-related HCC. **D** Alterations in metabolic pathways in the microbiota of HBV-related and non-HBV-related HCC based on the MetaCyc database

Previous reports have revealed that differences in tumour characteristics, such as pathological stages, could be attributed to remarkable differences in microbial infiltration in the TME. Herein, we analysed the relationship between the abundance of intratumorally infiltrated bacteria and the clinical characteristics of patients. Hepatitis B virus (HBV) infection persistently damages hepatocytes and causes inflammatory changes in the TME of HCC. We found that the infiltrated colonies were more diverse in the TME of HBV-related HCC tissues than in that of non-HBV-related HCC tissues (Fig. 6A, B). In particular, the abundances of *Dietzia* and *Oscillibacter* were lower in the HBV group, while those of *Veillonella* and *Alloprevotella* were higher (Fig. 6C). The metabolic function prediction results showed that the thiamin diphosphate biosynthesis pathway was inhibited in the HBV group, while the degradation of glutamate and the biosynthesis of diacylglycerol were significantly enhanced (Fig. 6D).

**Fig. 7:**
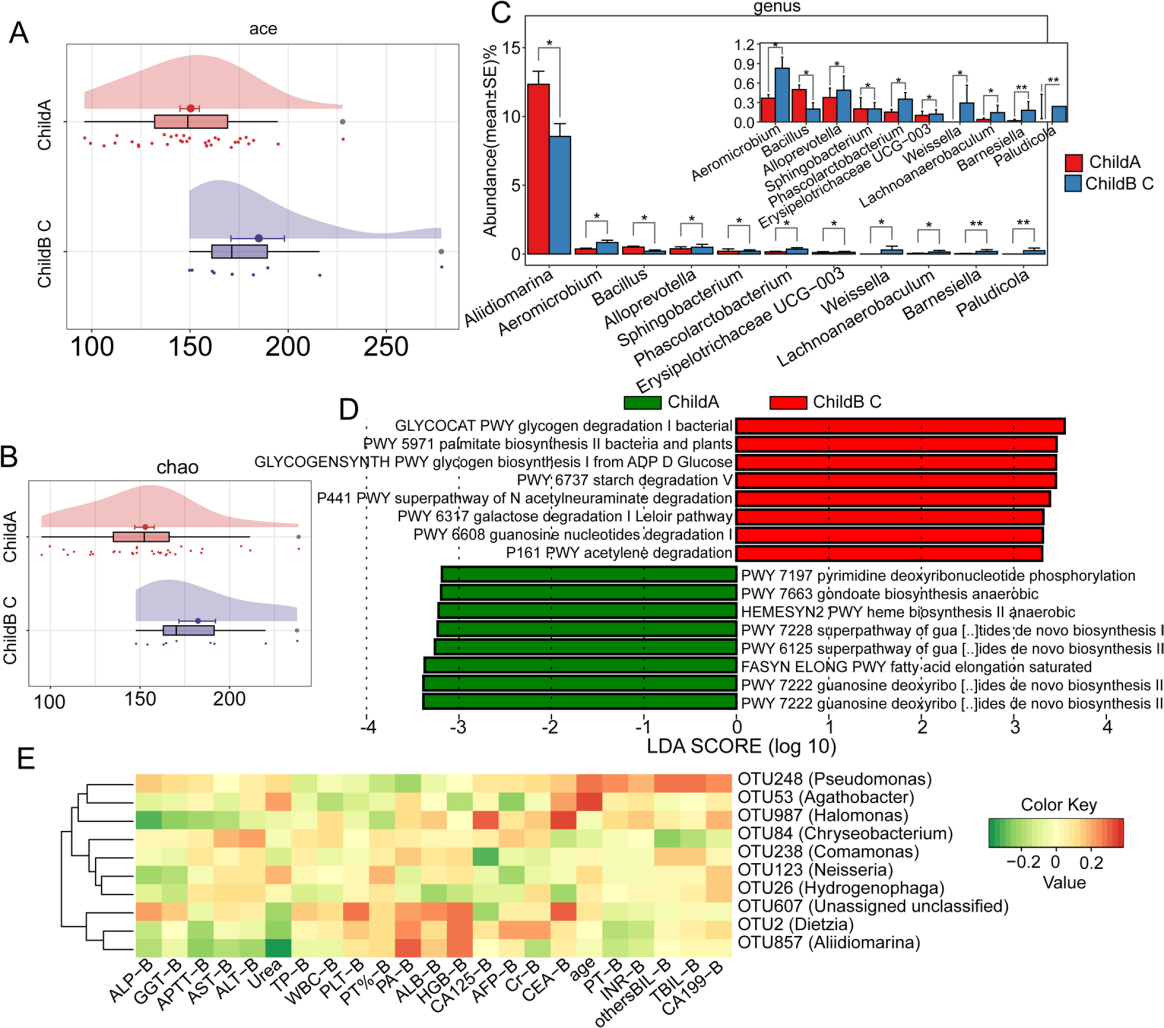
Differences in intrahepatic microbiota between HCC patients with Child‒Pugh A and Child‒Pugh B-C and their correlation with biochemical markers. **A**, **B** Differences in the Ace and Chao indices of microbiota between Child‒Pugh A and Child‒Pugh B-C HCC. **C** Microbial differences at the genus level between Child‒Pugh A and Child‒Pugh B-C HCC. **D** Alterations in metabolic pathways in the microbiota of Child‒Pugh A and Child‒Pugh B-C HCC based on the MetaCyc database. **E** Correlation analysis of biochemical indices and intratumoral microflora in HCC patients

The vast majority of HCCs originate from different degrees of liver cirrhosis. As a key indicator for evaluating the severity of liver cirrhosis, the Child‒Pugh score can be used to comprehensively estimate liver function. We found that the Child‒Pugh scores appeared to be strongly correlated with the extent of bacterial infiltration in the TME. Child‒Pugh B-C HCC patients exhibited significantly higher bacterial diversity than Child‒Pugh A HCC patients (Fig. 7A, B). At the genus level, the abundances of most microbes, such as *Aeromicrobium*, *Sphingobacterium* and *Erysipelotrichaceae*, were significantly higher in Child B-C HCC tissues, while the abundance of *Aliidiomarina* and *Bacillus* was lower (Fig. 7C). Functionally, the microbiota in this group of HCC tissues were found to play roles in processes such as glycogen metabolism and the degradation of molecules such as acetylneuraminic acid, galactose, and purines (Fig. 7D). Considering that patients with higher Child‒Pugh scores generally have symptoms such as ascites, intra-abdominal infection, and hepatic encephalopathy, the role of the microbiota and their metabolites deserves further investigation.

Subsequently, we evaluated the correlation of various preoperative clinical indicators of patients with bacterial abundance. We found that *Comamonas* abundance was significantly negatively correlated with the levels of CA125, whereas *Halomonas* abundance was significantly positively correlated with CA125 and CEA levels and negatively correlated with ALP levels (Fig. 7E). This finding further illustrates the influence of certain bacteria within the tumour, such as *Halomonas*, a bacterium that has been shown to be associated with cancer, as well as their metabolites on tumour cells. We comprehensively evaluated the differences in tumour microbial infiltration among samples obtained from patients with different tumour-node-metastasis (TNM) stage, tumour numbers, and Barcelona Clinic Liver Cancer (BCLC) stages (Additional file 1: Fig. S1). Interestingly, according to these results, the bacterial diversity within the tumour increases with tumour size/malignancy.

## Discussion

Interactions between microbes and the human body have been well documented; microbes can affect a variety of physiological functions, including metabolism and the immune system [17]. These interactions are also observed in cancer. Martel et al. suggested that at least 20% of cancers are influenced by the microbiota [18]. Therapeutic responsiveness (e.g., to immunotherapy and chemotherapy) has been observed to depend on the gut microecosystem in animal experiments and cancer patients [19–23]. Previous studies have focused on the interaction between gut microbes and specific diseases, and although multiple noninvasive biomarkers of particular diseases have been developed, due to the limitation that the microbiota play indirect roles in disease, the precise identification of the causal relationship between gut microbes and diseases, including cancer, remains a challenge [24, 25]. Intratumoral microbes play a more direct role on a more local scale than gut microbes to influence tumour progression [8]. Such microbes have been shown to interact with tumour cells in diseases such as lung, colorectal and skin cancers [11, 26–28]. Mechanistically, intratumoral microbes act through three principal mechanisms: (1) their metabolites regulate oncogenes or oncogenic pathways; (2) they promote DNA damage and gene mutation; and (3) they regulate immune responses in the microenvironment [8, 10, 11]. Overall, these effects inhibit the antitumour response. Nejman et al. demonstrated that intratumoral microbiota are tumour specific, which may imply that the metabolic pathways in which microorganisms are involved in different microenvironments can differentially affect tumour cells [9]. Therefore, to further clarify the mechanism underlying HCC progression, it is necessary to explore the intratumoral microbes associated with HCC.

In this study, we assessed the microenvironment of HCC and confirmed the presence of bacteria in immune cells using FISH technology. Specifically, we performed MiSeq sequencing using 99 HCC and paracancerous tissues based on 16S rRNA sequence technology, which yielded a comprehensive map of the microbes within HCC and paracancerous tissues. The results showed that the microbial community diversity, including alpha and beta diversity, was significantly higher in HCC tissues than in paracancerous tissues. However, the dominant species of the microflora (Proteobacteria, Firmicutes, Actinobacteria, etc.) did not differ between the two groups, but their levels were slightly different. We conjectured that certain microorganisms with low relative abundance may contribute more to the difference between the two groups. The significant elevations in *Enterobacteriaceae*, *Neisseria* and *Fusobacterium* abundance in tumour tissues caught our attention. A high abundance of *Enterobacteriaceae* is often associated with higher levels of inflammation, which may be related to the ability of this type of microorganism to utilize inflammatory byproducts (such as nitrate) in the microenvironment as energy sources, which is an ability that is not found in competing bacteria [29–31]. Moreover, virulence factors secreted by *E. coli*, such as cytolethal distending toxin (CDT), further aggravate the inflammatory response and directly induce DNA damage [32]. Considering that inflammation is a recognized risk factor for cancer, this may be a potential mechanism through which Enterobacteriaceae is involved in the progression of HCC [33]. Similarly, *Fusobacterium* plays a role in proinflammatory processes [34]. Moreover, a unique ability of *Fusobacterium* may be more involved in the progression of HCC: the bacterium can shuttle noninvasive bacteria into the cytoplasm of host cells [35]. Considering the direct contact that occurs between intratumoral microbes and tumour cells in the TME, this ability could theoretically have a greater influence. This indicates that even bacteria with levels that are not significantly different between HCC and paracancerous tissue can be involved in tumour progression by a mechanism that remains to be elucidated; uncovering this potential mechanism is obviously a great challenge to the pursuit of a full understanding of the interaction between intratumoral microbes and tumours. In addition, the increased abundance of certain tumour-promoting bacteria, such as *Neisseria,* and the low infiltration of antitumour bacteria, such as *Pseudomonas*, may further influence HCC progression [36, 37]. The research conducted by Rolandas Gedgaudas et al. proved that intestinal permeability in patients with portal hypertension was significantly higher than that in the healthy control group, which was accompanied by a high abundance of some bacteria in peripheral blood [38]. These bacteria mainly included Enterobacteriaceae, Shigella and many other kinds of bacteria, which is consistent with the high abundance of bacteria in HCC tissues observed in our study. This finding indicates that the progression of portal hypertension is accompanied by bacterial translocation, which can partly explain the source of intratumoral bacteria in HCC tissue.

Another interesting phenomenon is that functional enrichment analysis showed that the metabolic pathway in which microorganisms associated with HCC were involved in featured significant enhancement of fatty acid and lipid biosynthesis. It has been confirmed that changes in lipid metabolism occur in rapidly proliferating cancer cells [39]. Cancer cells transfer more carbon to fatty acids for membrane and signalling molecule biosynthesis than normal cells to maintain rapid cell growth [40]. Previous studies have shown that tumour cells tend to synthesize fatty acids de novo [41]. However, the entry of pyruvate into the TCA cycle is inhibited due to the hypoxic TME, and the resulting reduction in fatty acid synthesis may be compensated by the increased uptake of exogenous lipids [42, 43]. Based on our research, we speculate that microbial metabolites in the TME may provide another plausible source of fatty acids and lipids for cancer cells, which in turn promotes their proliferation and invasion.

Even within the HCC group, different clinical features led to differential microbial communities. For example, the abundance of *Veillonella* and *Alloprevotella* was significantly increased in the TME of HBV-related HCC tissues. Higher Child‒Pugh scores tended to favour the high abundance of *Sphingosineum* and *Erysipelas*. Based on the current data, we are not able to precisely interpret the relationship between changes in the abundance of specific bacteria and clinical features. Certainly, these microbes affect changes in the clinical characteristics of patients in their own unique ways. Collectively, the microbial community associated with HCC causally interacts with the unique TME, influencing the progression of HCC through multiple mechanisms. While we cannot accurately assess the relationship between the two due to the lack of related research, we believe that our findings provide a basis and guidance for further exploration in this field.

## Conclusion

The present study involved microbial profile analysis of human HCC tissue and attempted to decipher the functional roles of specific microbes in tumour progression. We found significant differences in microbial communities in HCC and paracancerous tissues. The high abundance of *Enterobacteriaceae* and *Fusobacterium* in HCC may affect HCC progression through various mechanisms.

## Supplementary Information

**Additional file 1. Figure S1** **A** Genus-level differences in intratumoral microbes between single and multiple tumors. **B** Differences in intratumoral microbes at the genus level among different T stages. **C** Differences in intratumoral microbes at the genus level among different BCLC stages. **D** Differences in intratumoral microbes at the genus level among different stages.

**Additional file 2. Table S1** OTU annotation of HCC and paracancerous tissue.

**Additional file 3. Table S2** Shared microbiome in HCC and paracancerous tissues at different sample coverages.

## Data Availability

The datasets generated and/or analysed during the current study are available from the corresponding author on reasonable request.

## References

[1] Bray F, Ferlay J, Soerjomataram I, Siegel RL, Torre LA, Jemal A (2018). Global cancer statistics 2018: GLOBOCAN estimates of incidence and mortality worldwide for 36 cancers in 185 countries. CA Cancer J Clin.

[2] Siegel RL, Miller KD, Jemal A (2020). Cancer statistics, 2020. CA Cancer J Clin.

[3] Llovet JM, Kelley RK, Villanueva A, Singal AG, Pikarsky E, Roayaie S, Lencioni R, Koike K, Zucman-Rossi J, Finn RS (2021). Hepatocellular carcinoma. Nat Rev Dis Primers.

[4] Valenti L, Pedica F, Colombo M (2022). Distinctive features of hepatocellular carcinoma in non-alcoholic fatty liver disease. Dig Liver Dis.

[5] Ruf B, Heinrich B, Greten TF (2021). Immunobiology and immunotherapy of HCC: spotlight on innate and innate-like immune cells. Cell Mol Immunol.

[6] Zhang DY, Friedman SL (2012). Fibrosis-dependent mechanisms of hepatocarcinogenesis. Hepatology.

[7] Garber K (2018). Driving T-cell immunotherapy to solid tumors. Nat Biotechnol.

[8] Garrett WS (2015). Cancer and the microbiota. Science.

[9] Nejman D, Livyatan I, Fuks G, Gavert N, Zwang Y, Geller LT, Rotter-Maskowitz A, Weiser R, Mallel G, Gigi E (2020). The human tumor microbiome is composed of tumor type-specific intracellular bacteria. Science.

[10] Dzutsev A, Badger JH, Perez-Chanona E, Roy S, Salcedo R, Smith CK, Trinchieri G (2017). Microbes and Cancer. Annu Rev Immunol.

[11] Ramirez-Labrada AG, Isla D, Artal A, Arias M, Rezusta A, Pardo J, Galvez EM (2020). The influence of lung microbiota on lung carcinogenesis, immunity, and immunotherapy. Trends Cancer.

[12] Edgar RC (2013). UPARSE: highly accurate OTU sequences from microbial amplicon reads. Nat Methods.

[13] Quast C, Pruesse E, Yilmaz P, Gerken J, Schweer T, Yarza P, Peplies J, Glockner FO (2013). The SILVA ribosomal RNA gene database project: improved data processing and web-based tools. Nucleic Acids Res.

[14] Wang Q, Garrity GM, Tiedje JM, Cole JR (2007). Naive Bayesian classifier for rapid assignment of rRNA sequences into the new bacterial taxonomy. Appl Environ Microbiol.

[15] Schloss PD, Westcott SL, Ryabin T, Hall JR, Hartmann M, Hollister EB, Lesniewski RA, Oakley BB, Parks DH, Robinson CJ (2009). Introducing mothur: open-source, platform-independent, community-supported software for describing and comparing microbial communities. Appl Environ Microbiol.

[16] Chen H, Liu Y, Zhang M, Wang G, Qi Z, Bridgewater L, Zhao L, Tang Z, Pang X (2015). A Filifactor alocis-centered co-occurrence group associates with periodontitis across different oral habitats. Sci Rep.

[17] Human Microbiome Project C (2012). Structure, function and diversity of the healthy human microbiome. Nature..

[18] de Martel C, Ferlay J, Franceschi S, Vignat J, Bray F, Forman D, Plummer M (2012). Global burden of cancers attributable to infections in 2008: a review and synthetic analysis. Lancet Oncol.

[19] Vetizou M, Pitt JM, Daillere R, Lepage P, Waldschmitt N, Flament C, Rusakiewicz S, Routy B, Roberti MP, Duong CP (2015). Anticancer immunotherapy by CTLA-4 blockade relies on the gut microbiota. Science.

[20] Viaud S, Saccheri F, Mignot G, Yamazaki T, Daillere R, Hannani D, Enot DP, Pfirschke C, Engblom C, Pittet MJ (2013). The intestinal microbiota modulates the anticancer immune effects of cyclophosphamide. Science.

[21] Sivan A, Corrales L, Hubert N, Williams JB, Aquino-Michaels K, Earley ZM, Benyamin FW, Lei YM, Jabri B, Alegre ML (2015). Commensal Bifidobacterium promotes antitumor immunity and facilitates anti-PD-L1 efficacy. Science.

[22] Gopalakrishnan V, Spencer CN, Nezi L, Reuben A, Andrews MC, Karpinets TV, Prieto PA, Vicente D, Hoffman K, Wei SC (2018). Gut microbiome modulates response to anti-PD-1 immunotherapy in melanoma patients. Science.

[23] Matson V, Fessler J, Bao R, Chongsuwat T, Zha Y, Alegre ML, Luke JJ, Gajewski TF (2018). The commensal microbiome is associated with anti-PD-1 efficacy in metastatic melanoma patients. Science.

[24] Vujkovic-Cvijin I, Sklar J, Jiang L, Natarajan L, Knight R, Belkaid Y (2020). Host variables confound gut microbiota studies of human disease. Nature.

[25] Walter J, Armet AM, Finlay BB, Shanahan F (2020). Establishing or exaggerating causality for the gut microbiome: lessons from human microbiota-associated rodents. Cell.

[26] Clay SL, Fonseca-Pereira D, Garrett WS (2022). Colorectal cancer: the facts in the case of the microbiota. J Clin Invest.

[27] Fu A, Yao B, Dong T, Chen Y, Yao J, Liu Y, Li H, Bai H, Liu X, Zhang Y (2022). Tumor-resident intracellular microbiota promotes metastatic colonization in breast cancer. Cell.

[28] Mrazek J, Mekadim C, Kucerova P, Svejstil R, Salmonova H, Vlasakova J, Tarasova R, Cizkova J, Cervinkova M (2019). Melanoma-related changes in skin microbiome. Folia Microbiol (Praha).

[29] Arthur JC, Perez-Chanona E, Muhlbauer M, Tomkovich S, Uronis JM, Fan TJ, Campbell BJ, Abujamel T, Dogan B, Rogers AB (2012). Intestinal inflammation targets cancer-inducing activity of the microbiota. Science.

[30] Carvalho FA, Koren O, Goodrich JK, Johansson ME, Nalbantoglu I, Aitken JD, Su Y, Chassaing B, Walters WA, Gonzalez A (2012). Transient inability to manage proteobacteria promotes chronic gut inflammation in TLR5-deficient mice. Cell Host Microbe.

[31] Winter SE, Winter MG, Xavier MN, Thiennimitr P, Poon V, Keestra AM, Laughlin RC, Gomez G, Wu J, Lawhon SD (2013). Host-derived nitrate boosts growth of *E. coli* in the inflamed gut. Science..

[32] Nesic D, Hsu Y, Stebbins CE (2004). Assembly and function of a bacterial genotoxin. Nature.

[33] Elinav E, Nowarski R, Thaiss CA, Hu B, Jin C, Flavell RA (2013). Inflammation-induced cancer: crosstalk between tumours, immune cells and microorganisms. Nat Rev Cancer.

[34] Dharmani P, Strauss J, Ambrose C, Allen-Vercoe E, Chadee K (2011). Fusobacterium nucleatum infection of colonic cells stimulates MUC2 mucin and tumor necrosis factor alpha. Infect Immun.

[35] Xu M, Yamada M, Li M, Liu H, Chen SG, Han YW (2007). FadA from Fusobacterium nucleatum utilizes both secreted and nonsecreted forms for functional oligomerization for attachment and invasion of host cells. J Biol Chem.

[36] Xie X, Yang M, Ding Y, Chen J (2017). Microbial infection, inflammation and epithelial ovarian cancer. Oncol Lett.

[37] Chang L, Xiao W, Yang Y, Li H, Xia D, Yu G, Guo X, Guan W, Hu Z, Xu H (2014). Pseudomonas aeruginosa-mannose-sensitive hemagglutinin inhibits epidermal growth factor receptor signaling pathway activation and induces apoptosis in bladder cancer cells in vitro and in vivo. Urol Oncol.

[38] Gedgaudas R, Bajaj JS, Skieceviciene J, Varkalaite G, Jurkeviciute G, Gelman S, Valantiene I, Zykus R, Pranculis A, Bang C (2022). Circulating microbiome in patients with portal hypertension. Gut Microbes.

[39] Santos CR, Schulze A (2012). Lipid metabolism in cancer. FEBS J.

[40] DeBerardinis RJ, Thompson CB (2012). Cellular metabolism and disease: what do metabolic outliers teach us?. Cell.

[41] Ookhtens M, Kannan R, Lyon I, Baker N (1984). Liver and adipose tissue contributions to newly formed fatty acids in an ascites tumor. Am J Physiol.

[42] Kamphorst JJ, Cross JR, Fan J, de Stanchina E, Mathew R, White EP, Thompson CB, Rabinowitz JD (2013). Hypoxic and Ras-transformed cells support growth by scavenging unsaturated fatty acids from lysophospholipids. Proc Natl Acad Sci USA.

[43] Bensaad K, Favaro E, Lewis CA, Peck B, Lord S, Collins JM, Pinnick KE, Wigfield S, Buffa FM, Li JL (2014). Fatty acid uptake and lipid storage induced by HIF-1alpha contribute to cell growth and survival after hypoxia-reoxygenation. Cell Rep.

