# Supplementary figures and images for: Overview of microbial profiles in human hepatocellular carcinoma and adjacent nontumor tissues
Source: J Transl Med. 2023 Feb 2;21:68. doi: 10.1186/s12967-023-03938-6 (PMC9893660; doi:10.1186/s12967-023-03938-6)

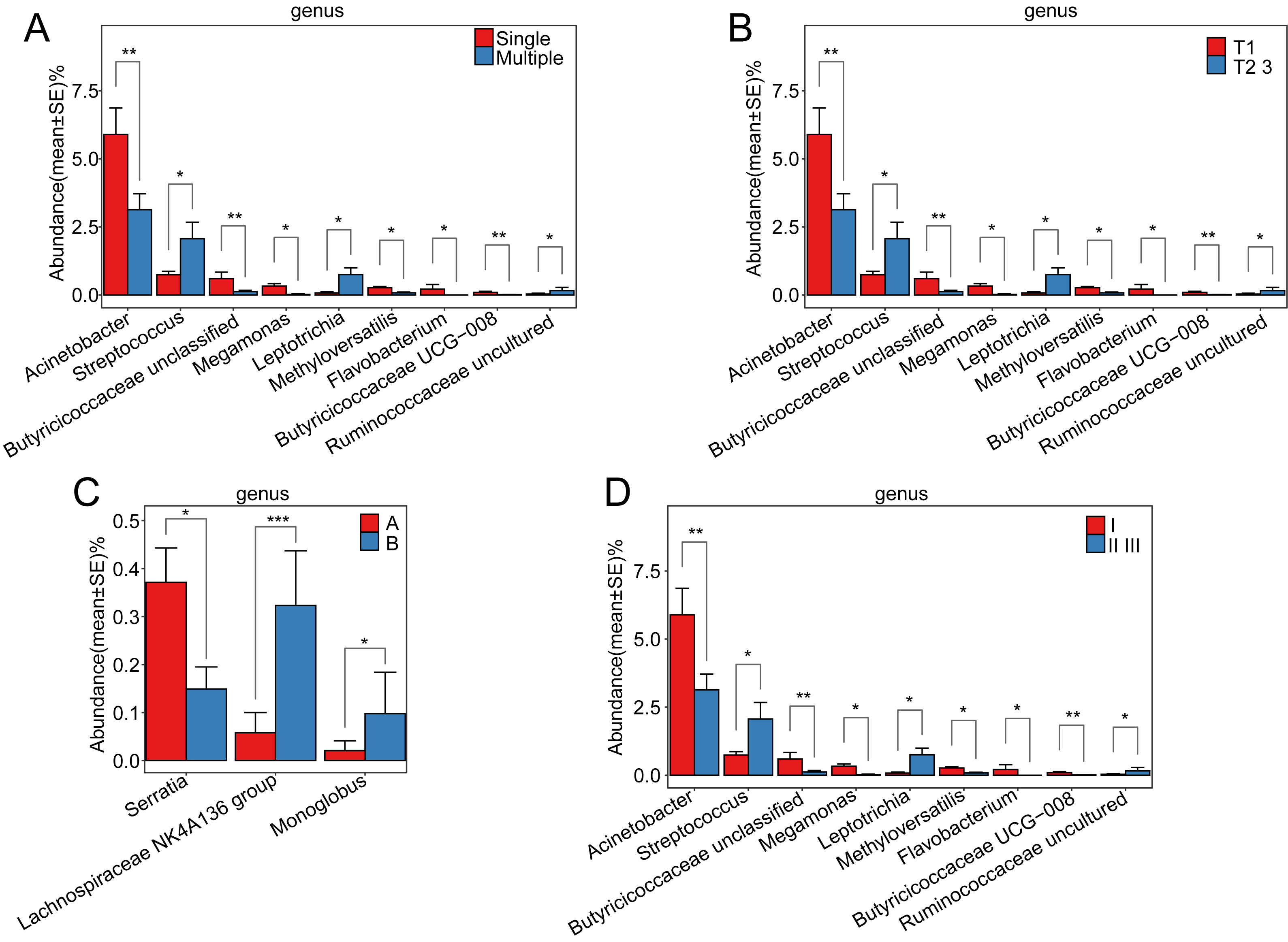

Supplement: Supplementary file 1 — Additional file 1. Figure S1 A Genus-level differences in intratumoral microbes between single and multiple tumors. B Differences in intratumoral microbes at the genus level among different T stages. C Differences in intratumoral microbes at the genus level among different BCLC stages. D Differences in intratumoral microbes at the genus level among different stages. [file 12967_2023_3938_MOESM1_ESM.tif]
